# 

**Published:** 1911-09-09

**Authors:** 


### BOOKS RECEIVED.

Percy Linley.
"On the East Coast." By the Great Eastern Railway.

John Wright and Sons, Ltd.
"Ship's Hygiene." By W. Melville-Davison.

John Ouseley, Ltd.
"Health and Empire." By Francis Fremantle.

H. K. Lewis.
"Diseases of the Lungs." By Sir R. Douglas Powell, Bart., and P. Horton-Smith Hartley, M.D.

Paul, Trench, Trubner and Co.
"Crystals." By A. E. H. Lutton.

J. and A. Churchill.

"Sight Testing Made Easy." W. W. Hardwicke.

### BUILDINGS CONTEMPLATED AND IN PROGRESS.

Gateshead.—An estimate amounting to £76,600 has been recommended for acceptance by the Town Council for the erection of the superstructure at the asylum. Messrs. Hive and Pegg, of London, are the architects.

Glasgow.—Important extensions to the Glasgow Western Infirmary are nearing completion. Additional ward accommodation costing £25,000 and a clinical laboratory costing £5,000 are the items, and a further extension scheme, including the construction of a reception ward with operating theatres over, is on foot.

Newcastle.—The Newcastle Board of Guardians have decided to erect an additional hospital block within the workhouse grounds, at an estimated cost of £18,000.

Perth.—Mr. Jas. Miller, A.R.S.A., of Glasgow, is architect for the new City and County Infirmary; the cost of the buildings is £36,190, and they will consist of four two-storey ward blocks with a centrally situated administrative block connected to the wards by a corridor. Each of the main wards will contain twelve beds, and in addition a single and double bedded room on each floor. Thirty-two single rooms are provided for nurses.

Reading.—Mr. W. R. Howell, A.R.I.B.A., is architect for the works at Reading Poor Law Buildings, costing £13,738. The work includes additions to the administrative block, a new operating-room, a new block for aged infirm, and alterations to the master's house.

York.—The Secretary of State for War has invited tenders for extensive alterations and additions to the military hospital in York; a new ward and operating theatre are included in the extension.

---

Under the will of Mrs. Sarah Pierce, of Whalley Range, Manchester, £1,000 is left to the Manchester Royal Infirmary and £500 each to the Hospital for Consumption and to the Hospital for Cancer in Manchester.

### MEDICAL APPOINTMENTS.

The following appointments were made last week by the Board of the Manchester Royal Infirmary: Mr. J. P. Buckley, F.R.C.S., to be Assistant Surgical Officer; Mr. R. C. Hutchinson, M.B., Ch.B., Vict., to be Medical Officer at the Central Branch; and Mr. Wm. Stirling, jun., M.B., Ch.B. Vict., to be Assistant Medical Officer at the Barnes Convalescent Hospital. The new House Physicians are: Mr. A. H. Holmes, M.B., Ch.B., Vict.; Mr. R. Briercliffe, M.B., Ch.B., Vict.; and Mr. G. E. Sawdon, M.B., Ch.B., Vict. The Senior House Surgeons are: Mr. J. Gow, M.B., Ch.B. Vict., and Mr. J. F. Cocker, M.B., Ch.B., Vict.; and the Junior House Surgeons are: Mr. W. A. Sneath, M.B., Ch.B. Vict.; Mr. F. Grey, M.B., Ch.B. Vict., and Mr. M. Moritz, M.B., Ch.B. Vict.; while Miss M. May, M.B., Ch.B., Vict., has been appointed qualified clinical assistant.

### THE WEEK'S NOVELTIES.

### AN ADJUNCT TO PATIENTS ON BALCONIES.

This exceptionally hot summer has brought to provincial and cottage hospitals, and sanatoria more particularly, face to face with the problem of how to protect patients in the open-air and on balconies from the bites of insects. We have received samples of a preparation called Muscatol, which is manufactured by Frank Rogers, of 327 Oxford Street, who claims for it absolute protection for patients from mosquitoes, midges, harvesters, and sandflies. A pocket bottle has been designed, which should be especially useful to patients lying on balconies beyond the immediate reach of protection from insects.

### APPOINTMENTS VACANT.

Full particulars of these vacancies can be obtained on reference to our advertisement columns:

Bradford Royal Infirmary.—House Physician and two House Surgeons.

Carlisle Dispensary.—Resident Medical Officer.

Cornwall County Asylum, Bodmin.—Third Assistant Medical Officer.

Leicester Infirmary.—House Physician.

Mullingar District Lunatic Asylum.—Resident Assistant Medical Officer.

Queen's Hospital for Children.—House Surgeon.

Raratonga, Cook Islands.—Assistant Medical Officer.

Royal Surrey County Hospital, Guildford.—Assistant House Surgeon.

Wolverhampton General Hospital.—House Surgeon.

